# An improved equation of latitude and a global system of graticule distance coordinates

**DOI:** 10.1007/s00190-023-01815-0

**Published:** 2024-01-08

**Authors:** Geoffrey Blewitt

**Affiliations:** Nevada Bureau of Mines and Geology, University of Nevada, 1664 N Virginia St, MS 178, Reno, NV 89557 USA

**Keywords:** Coordinate systems, Geodetic coordinates, Equation of latitude, Coordinate transformations, Time series, Global geodesy, Geodetic displacements

## Abstract

Two innovations are presented for coordinate time-series computation. First, an improved solution is given to a century-old problem, that is the non-iterative computation of conventional geodetic (CG: latitude, longitude, height) coordinates from geocentric Cartesian (GC: *x*, *y*, *z*) coordinates. The accuracy is 1 nm for heights < 500 km and < 10^−15^ rad for latitude at any point, terrestrial or outer space. This can be 3 orders of magnitude more accurate than published non-iterative methods. Secondly, CG time series are transformed into a practical system of “graticule distance” (GD: easting, northing, height) curvilinear coordinates that, unlike the commonly used system of topocentric Cartesian (TC: east, north, up) coordinates, is global in nature without arbitrary specification of GC reference coordinates for every geodetic station. Since 2011, Nevada Geodetic Laboratory has publicly produced time series for 20,000 GPS stations in GD form that have been cited by hundreds of studies. The GD system facilitates direct comparison of positions for co-located stations. Users of GD time series are able: (1) to resolve different historical station names that have been assigned to the same physical benchmark and (2) to resolve different physical benchmarks that have been assigned the same name. This benefits historical reconstruction of benchmark occupation and local site tie analysis for reference frame integrity. GD coordinates have archival value, in that inversion back to GC coordinates is practically exact. For geodetic stations, GD time series closely emulate TC time series with rates agreeing to 0.01 mm/yr, and so can be used interchangeably.

## Introduction

A major objective of modern space geodesy is to provide geophysical users with precise time series of station positions in a global conventional reference frame that is sufficiently stable to determine accurate displacements of the Earth’s surface on time scales of seconds to multiple decades. Here we present two innovations in the computation of station coordinate time series that are of proven benefit to both producers and users of geodetic time series:

1. The first innovation involves a new non-iterative method to compute latitude, a problem with a century-old history. The method presented here has a computational accuracy of 1 nm for near Earth locations, which can be 3 orders of magnitude better than commonly adopted non-iterated methods (Bowring 1976; Bowring 1985).
2. The second innovation is the development of a new global system of coordinates with displacements that closely approximate that of the usual topocentric (east, north, up) coordinate systems local to each geodetic station. The global nature of this system enables new practical capabilities for co-located stations; hence, the practical aspects are important to describe here, as well as the theoretical aspects.

The current general procedure in space geodesy (Seeber 1993) starts by estimating geodetic station positions as geocentric Cartesian (GC) coordinates $(x,y,z)$, with traceability to the International Terrestrial Reference System (IERS Conventions 2010). Station positions are then be transformed into conventional geodetic (CG) reference coordinates, latitude $\phi$, longitude $\lambda$, and height, $h$. Next, the GC coordinate time series for each station are transformed into topocentric Cartesian (TC) coordinates $(e, n, u)$ with orthogonal coordinate axes aligned with the local east, north, and up directions (Seeber 1993). This procedure requires GC reference coordinates $\left({x}_{0},{y}_{0},{z}_{0}\right)$ and CG reference coordinates $\left({\phi }_{0},{\lambda }_{0}\right)$ for each station, for example, at the first epoch of the time series.

There are several reasons for this conversion from global to local coordinates: (1) errors in $(e, n, u)$ are generally much less correlated than $(x,y,z)$, thus facilitating interpretation of time series for individual components; (2) formal errors and observed scatter of time-series coordinates is generally lower in east and north versus the up component; (3) geophysical modeling and interpretation is usually simpler in the TC system as many geophysical processes naturally separate into horizontal and vertical displacements (e.g., tectonic plate motions and surface loading), and sometimes into east and north components (e.g., tropospheric and ionospheric delay).

As an alternative to TC coordinates, presented here is a global system of “graticule distance” (GD) coordinates. GD coordinates $(E, N, h)$ include easting $E$(m), northing $N$(m), and conventional ellipsoidal height $h$(m). GD coordinates $(E, N, h)$ map uniquely to locations relating to the surface of the conventional GRS80 ellipsoid (Moritz 2000) and can be inverted back to GC coordinates $(x,y,z)$ to within 2 nm, which is practically exact.

Displacements of geodetic stations in GD $\left(E, N,h\right)$ coordinates are designed to closely track those of TC $(e, n, u)$ coordinates. Geophysical users switching from TC to GD station coordinates would not notice any significant difference in displacement time series of geodetic stations. Their differences grow with increasing displacement owing to Earth curvature being incorporated in the GD curvilinear axes, but not in the linear axes of the TC system. Coordinate displacements in either system typically track each other to within 0.1 mm for stations subject to geophysical motion. These differences can generally be ignored for investigations of Earth processes, such as in tectonics, plate boundary deformation, earthquakes, and surface mass loading. In general, both TC and GD coordinates can be treated equivalently and can be accurately interpreted as horizontal and vertical displacements for local areas, though such interpretation tends to break down for displacements much greater than 10 m.

Importantly, in contrast to the TC system, GD coordinates $(E, N, h)$ are computed directly from each station’s GC coordinates $(x,y,z)$ without need for different station-specific reference positions $\left({x}_{0},{y}_{0},{z}_{0}\right)$. This gives a practical advantage to the GD system, in that coordinates from locally neighboring stations can be compared directly. This helps detective work into metadata problems, such as (1) whether historical stations of different names occupied the same benchmark, perhaps centered but with a different height, and (2) whether station data sets of the same name are actually on different benchmarks that can be identified.

The structure of this paper starts by presenting a new non-iterative method to compute CG coordinates $(\phi , \lambda , h)$, which gives a practically exact closed-form solution for Earth-bound positions. Development of this new method stands on its own as an innovation in geodesy. It also allows us efficiently to examine the true accuracy of GD computation and its inversion back to GC coordinates without any impact of error from the CG computation.

Next, the new GD system is presented in detail, where all computations are specified. Inverse computations are also specified. Then results are presented, which show (1) how well inverse computations on GD time series reproduce the original GC time series, (2) how well in practice GD time-series displacements reproduce TC displacements. Focus is given to extreme cases where either system may break down for theoretical reasons, such as being close to the coordinate singularities at the poles, or where total displacements are relatively large for geophysical processes.

Finally, this leads to a discussion on the benefits of the GD system. GD coordinates have been implemented and used by Nevada Geodetic Laboratory (NGL) since 2011 to publish publicly available geodetic time series, currently for 20,000 GPS stations, which have been cited by hundreds of refereed journal articles (Blewitt et al. 2018). Practical examples of GD benefits are given based on user experience documented in the literature.

## Methods

The computational methodology described in this section aims to be self-contained to enable full reproducibility when using double precision numbers. Included here are all the equations required to reproduce the computation of CG $(\phi , \lambda , h)$ and GD $(E, N, h)$ starting with GC $(x,y,z)$.

This paper uses the following GRS80 ellipsoid parameters (Moritz 2000):

1 $$\begin{array}{l}\text{equatorial radius}=a=6 378 137 {\text{m}}\\ {\text{flattening}}=f=0.00\text{3 352 810 681 183 637 418}\end{array}$$

Note that flattening is a “derived parameter” in GRS80 and can be computed with arbitrary precision. For convenience, the above flattening has number of digits exceeding the published value (Moritz 2000) so that it well exceeds the double precision limit.

In addition, we use the following derived parameters:

2 $$ \begin{array}{*{20}l}    {{\text{polar}}\;{\text{radius}} = b = a\left( {1 - f} \right)} \hfill  \\    {{\text{first}}\;{\text{eccentricity}}\;{\text{squared}} = e^{2}  = \frac{{a^{2}  - b^{2} }}{{a^{2} }} = f\left( {2 - f} \right)} \hfill  \\    {{\text{second}}\;{\text{eccentricity}}\;{\text{squared}} = e^{{\prime 2}}  = \frac{{a^{2}  - b^{2} }}{{b^{2} }} = \frac{1}{{\left( {1 - f} \right)^{2} }} - 1} \hfill  \\   \end{array}  $$

We also use the following coordinate functions:

3 $$ \begin{gathered} {\text{polar}}\;{\text{axis}}\;{\text{distance}}\;p = \sqrt {x^{2} + y^{2} } \hfill \\ {\text{geocentric}}\;{\text{distance}}\;r = \sqrt {p^{2} + z^{2} } \hfill \\ \end{gathered} $$

### Computation of CG $(\phi , \lambda , h)$ from GC $(x,y,z)$

At every epoch, we need to convert GC coordinates $\left(x,y,z\right)$ to conventional geodetic (CG) coordinates, $(\phi , \lambda , h)$. The computation therefore needs to be efficient, with sufficient accuracy so as not to limit that of the GD coordinates.

Going the other direction, the forward transformation from GC to CG is exact (Torge 1980):

4 $$ \begin{aligned} x &= \left[ {R_{N} \left( \phi \right) + h} \right]\cos \phi \cos \lambda \hfill \\ y &= \left[ {R_{N} \left( \phi \right) + h} \right]\cos \phi \sin \lambda \hfill \\ z &= \left[ {\left( {1 - e^{2} } \right)R_{N} \left( \phi \right) + h} \right]\sin \phi \hfill \\ \end{aligned} $$

where radius of curvature of the prime vertical ${R}_{N}\left(\phi \right)$ is given by:

5 $${R}_{N}\left(\phi \right)=\frac{a}{\sqrt{1-{e}^{2}{{\text{sin}}}^{2}\phi }}$$

We seek the inverse transformation from CG to GC. First, from the above, the exact equation of longitude is clear:

6 $$ \begin{gathered}   {\text{Equation}}\;{\text{of}}\;{\text{longitude}} \hfill \\   \lambda  = \arctan \left( {\frac{y}{x}} \right) \hfill \\  \end{gathered}  $$

As advocated by Vincenty (1975), this should be implemented using programming function ${\text{ATAN}}2\left(y,x\right)$ so that (1) the correct quadrant is determined using signs of the arguments and (2) there is no singularity at $x=0$. Note that the order of arguments for the ${\text{ATAN}}2$ function depends on programming language; for example, the convention ${\text{ATAN}}2\left(x,y\right)$ typically holds for spreadsheets.

Next, we need to compute latitude and height. There is a plethora of articles (e.g., Hirvonen and Moritz 1963; Bowring 1976, 1985; Vermeille 2002) and textbooks (Bomford 1971; Torge 1980; Seeber 1993) on methods to disentangle latitude and height. An iterative method was suggested by Hirvonen and Moritz (1963), who (like others) acknowledge the computation is “rather complicated”. The method starts with two equations for height as a function of latitude, which at this point of the computation is not known:

7 $$h={h}_{1}=\frac{p}{{\text{cos}}\,\phi }-{R}_{N}$$

8 $$h={h}_{2}=\frac{z}{{\text{sin}}\,\phi }-{R}_{N}+{e}^{2}{R}_{N}$$

Hirvonen and Moritz (1963) difference the above equations, which achieves the goal of separating latitude from height, but is not the most efficient method for purposes of iteration. Instead, dividing the two equations gives the more modern textbook equation for latitude as a function of height (Torge 1980):

9 $${\text{tan}}\,\phi =\frac{z}{p}{\left[1-{e}^{2}\frac{{R}_{N}}{{R}_{N}+h}\right]}^{-1}$$

This textbook method iterates by initializing latitude assuming zero height, then radius of curvature and height are computed using latitude, and so on. One problem with this method is that the equations for height are either singular at the poles or the equator, depending on the choice of ${h}_{1}$ or ${h}_{2}.$

Instead, NGL implements the following equation (Bowring 1985), which uses a trigonometrical identity to combine Eqs. (7) and (8):

10 $$ \begin{aligned} h &= h_{1} {\text{cos}}^{2} \phi + h_{2} \sin^{2} \phi \hfill \\ h &= p\cos \phi - R_{N} {\text{cos}}^{2} \phi + z\sin \phi - R_{N} \sin^{2} \phi + e^{2} R_{N} \sin^{2} \phi \hfill \\ h &= p\cos \phi + z\sin \phi - a\sqrt {1 - e^{2} \sin^{2} \phi } \hfill \\ \end{aligned} $$

This equation removes the singularities and proves to be orders of magnitude more numerically accurate than either of Eqs. (7) and (8) of Hirvonen and Moritz (1963). Bowring (1985) notes that Eq. (10) is relatively insensitive to errors in latitude, which is a critical property exploited here for the equation of latitude.

Non-iterative methods have been proposed to separate latitude from height (e.g., Borkowski 1989; Vermeille 2002). Various studies have performed comparative tests of the many iterative and non-iterative methods. For example, in an extensive comparison, Claessens (2019) recommends Eq. (10) for the equation of height, together with the commonly adopted equation of latitude by Bowring (1976):

11 $${\text{tan}}\;\phi =\frac{z+{e^{\prime}}^{2}b{{\text{sin}}}^{3}U}{p-{e}^{2}a{{\text{cos}}}^{3}U}$$

where $U$ is reduced latitude (or parametric latitude) defined by

12 $${\text{tan}}\,U=\left(1-f\right){\text{tan}}\,\phi $$

For heights $h<{e}^{2}a=43 \text{ km}$, Bowring (1976) recommends approximating $U$ to reduced latitude on the surface of the ellipsoid:

13 $${\text{tan}}\,U\approx \frac{z}{\left(1-f\right)p}$$

Although technically this method might be iterative, in practice it is often implemented without iteration, which is why the method is widely adopted.

Alternatively, Bowring (1985) suggested a different setting for $U$ as follows, here given in the form by Soler and Hothem (1989):

14 $${\text{tan}}\,U\approx \frac{z}{p}\left(1-f+\frac{{e}^{2}a}{r}\right)$$

The motivation for this was to reduce dependence of errors on height and reduce errors in outer space; however, with such a compromise, it tends to underperform Bowring (1976) for Earth-bound heights.

Here, an improved setting for $U$ is presented which gives no detectable error for double precision computation of latitude for any point, terrestrial or outer space, without iteration. This proves to be 3 orders of magnitude more accurate than either Bowring (1976) or Bowring (1985), without iteration. The CPU time is increased by a modest 5%.

It can be shown that the tangent of reduced latitude can be written exactly in the following form:

15 $${\text{tan}}\,U=\frac{z}{p}\frac{\left(1-f\right)}{\left(1-\frac{{e}^{2}a}{{D}_{\text{exact}}}\right)}$$

where

16 $${D}_{\text{exact}}=a+h\sqrt{1-{e}^{2}{{\text{sin}}}^{2}\phi }$$

Given that $h$ is unknown, we proceed using the approximation:

17 $${D}_{\text{exact}}\approx D=r+f{\left(\frac{z}{r}\right)}^{2}\left(2a-r\right)$$

This approximation is now derived. The equation for ${D}_{\text{exact}}$ can be rewritten exactly by substituting $h$ given by Eq. (10) into Eq. (16):

18 $$ \begin{aligned} D_{{{\text{exact}}}} & = a + \left( {p\cos \phi + z\sin \phi - a\sqrt {1 - e^{2} \sin^{2} \phi } } \right)\sqrt {1 - e^{2} \sin^{2} \phi } \\ & = \left( {p\cos \phi + z\sin \phi } \right)\sqrt {1 - e^{2} \sin^{2} \phi } + e^{2} a\sin^{2} \phi \\ \end{aligned} $$

This is Taylor-expanded to first order in flattening $f\approx {e}^{2}/2$

19 $$ \begin{aligned} D_{{{\text{exact}}}} \approx D & = \left( {p\frac{p}{r} + z\frac{z}{r}} \right)\left( {1 - f\left( \frac{z}{r} \right)^{2} } \right) + 2fa\left( \frac{z}{r} \right)^{2} \\ & = r + f\left( \frac{z}{r} \right)^{2} \left( {2a - r} \right) \\ \end{aligned} $$

Hence, by substituting Eq. (19) into Eq. (15), the following approximation is obtained for tangent of reduced latitude:

20 $${\text{tan}}\;U\approx \frac{z}{p}\frac{\left(1-f\right)}{\left(1-\frac{{e}^{2}a}{ r+f{\left(\frac{z}{r}\right)}^{2}\left(2a-r\right)}\right)}$$

The reduced latitude from Eq. (20) is then inserted into Eq. (11) to compute latitude. The following shows explicitly the sequence of steps for computing the equation of latitude, which avoids inefficient trigonometric functions except at the very last step.

21 $$ \begin{gathered} {\text{Equation}}\;{\text{of}}\;{\text{latitude}} \hfill \\ P = \frac{p}{1 - f}\left( {1 - \frac{{e^{2} a}}{{r + f\left( \frac{z}{r} \right)^{2} \left( {2a - r} \right)}}} \right) \hfill \\ R = \sqrt {P^{2} + z^{2} } ; C = \frac{P}{R}; S = \frac{z}{R}; \hfill \\ T = \frac{{z + e^{{\prime}{2}} bS^{3} }}{{p - e^{2} aC^{3} }}{ ;} \phi = \arctan \left( T \right) \hfill \\ \end{gathered} $$

The following shows explicitly the steps for computing the equation of height, without need for trigonometric functions, starting with the results for $T$ from equation of latitude (21).

22 $$ \begin{gathered} {\text{Equation}}\;{\text{of}}\;{\text{height}} \hfill \\ C = \frac{1}{{\sqrt {1 + T^{2} } }}; S = TC; \hfill \\ h = pC + zS - a\sqrt {1 - e^{2} S^{2} } \hfill \\ \end{gathered} $$

### Computation of TC $(e, n, u)$ from GC $(x,y,z)$

In this paper, TC coordinates $(e, n, u)$ are only used as a basis of comparison with GD coordinates $(E, N, h)$ that will be described in Sect. 2.3. The standard equations are given here for completeness and to ensure reproducibility. To compute them, first, a reference triplet of geocentric Cartesian coordinates $\left({x}_{0},{y}_{0},{z}_{0}\right)$ is selected, which can be the first epoch coordinates in a time series, or any other point within 30 m of the triplets comprising the time series. This is required to keep the effects of Earth curvature < 0.1 mm.

The transformation into topocentric coordinates is given by (e.g., Seeber 1993):

23 $$\left(\begin{array}{l}e\\ n\\ u\end{array}\right)=\mathbf{R}\left(\begin{array}{c}x-{x}_{0}\\ y-{y}_{0}\\ z-{z}_{0}\end{array}\right)$$

The rotation matrix is:

24 $$\mathbf{R}=\left(\begin{array}{lll}\quad\;-{\text{sin}}\;{\lambda }_{0}& \quad\;\;{\text{cos}}\;{\lambda }_{0}& \quad0\\ -{\text{sin}}\;{\phi }_{0}\;{\text{cos}}\;{\lambda }_{0}& -{\text{sin}}\;{\phi }_{0}\;{\text{sin}}\;{\lambda }_{0}& {\text{cos}}\;{\phi }_{0}\\ \;\;\;{\text{cos}}\;{\phi }_{0}\;{\text{cos}}\;{\lambda }_{0}& \;\;\;{\text{cos}}\;{\phi }_{0}\;{\text{sin}}\;{\lambda }_{0}& {\text{sin}}\;{\phi }_{0}\end{array}\right)$$

where CG reference coordinates $\left({\phi }_{0},{\lambda }_{0}\right)$ are computed from the GD reference coordinates $\left({x}_{0},{y}_{0},{z}_{0}\right)$, in our case, using the method of Sect. 2.1, Eqs. (6) and (21).

The inverse transformation is given by:

25 $$\left(\begin{array}{l}x-{x}_{0}\\ y-{y}_{0}\\ z-{z}_{0}\end{array}\right)={\mathbf{R}}^{{\text{T}}}\left(\begin{array}{c}e\\ n\\ u\end{array}\right)$$

This inverse transformation is useful, for example, if the assumed antenna height requires correction in GC coordinates.

### Graticule distance (GD) coordinate $(E, N, h)$ system definition

GD horizontal coordinates $(E, N, h)$ are defined as graticule arc distances on the surface of the ellipsoid from each coordinate origin. The horizontal system $(E, N)$ is equivalent to geodetic longitude and latitude $(\lambda ,\phi )$ except that coordinate units are of distance rather than angle.

1. There are 3601 origins of easting, or “reference longitudes” ${\lambda }_{0}(n)$, spaced apart by 0.1 degrees (~ 11 km on the equator). The zone boundaries are halfway between reference longitudes. Starting from longitude of –180 degrees and moving eastward, the first reference longitude is at -180 degrees, the second at –179.9 degrees, and so on, until we reach the last origin at + 180 degrees. Zones are numbered from –1800 to + 1800. Theoretically, the first and last zones are identical, but in practice, the sign of the zone is determined by the sign of longitude of the station. Easting $E$ is negative west of the reference longitude, and positive east of the reference longitude. Easting is the distance from the reference longitude on the surface of the ellipsoid computed on an arc of constant latitude. Note two systematic effects on easting: (1) easting is ill-defined close to the coordinate singularities at the poles, so one question is whether this is significant in practice for real stations in polar regions; (2) if a station has a northward displacement, the latitude will change, and the easting will get slightly smaller in this scheme as meridians come closer together in units of distance. Such an effect controls the design as to the maximum width of a reference longitude zone. These systematic effects will be investigated in Sect. 3.5 and 3.6.
2. The origin of northing is the equator ${\phi }_{0}=0$. Northing $N$ is positive in the northern hemisphere and negative in the southern hemisphere. The magnitude of northing is the meridional distance from the equator, for which the method here uses the formulas by Vincenty (1975). Numerical integration (Sect. 2.6) is used to check the accuracy of the method (Sect. 3.4).
3. Height is the usual conventional geodetic height; that is, the origin is the surface of the ellipsoid, and height is the normal signed distance from the ellipsoid to the station. Height is positive above the ellipsoid and negative below the ellipsoid.

### Computation of easting $E(\phi ,\lambda )$

First identify the zone number $n$, where longitude $\lambda$ is in units of degrees ranging from $-180$ to $+180$, and “nint” is the nearest integer function:

26 $$n={\text{nint}}\left(10\lambda \right)$$

Compute the reference longitude in degrees:

27 $${\lambda }_{0}(n)=0.1n$$

Compute the easting $E$, taking care to convert degrees into radians:

28 $$E\left(\phi ,\lambda \right)=\frac{\pi }{180}\left[\lambda -{\lambda }_{0}(n)\right] {R}_{N}\left(\phi \right){\text{cos}}\phi $$

Note that easting is a function of the zone number $n$. It is desirable to retain the same zone number $n$ for the rare case of a station crossing into the next zone $n\pm 1$ so that the easting time series does not have an inconvenient (through exactly known) discontinuity. In such a case, for example, the same zone could be retained if the change in easting between epochs is less than 10 m. Otherwise, if the change is greater than 10 m or if the zone is not a nearest neighbor, the easting changes its zone and reference longitude. To implement this requires the time series computation to save the last values of the zone number and easting.

Changing the zone number is typically necessary if, by blunder, two stations happen to have the same name, and the data get mixed up in the same time series. In practice, this can happen surprisingly often. Implementing the code in this way enhances diagnosis of blunders.

### Computation of northing $N(\phi )$

There is a long history of published equations for the computation of meridional distance from the equator, many of which would be equivalent to the method used in this paper. Our method is given explicitly so that results can be reproduced. Here, the equation for precise computation of northing $N$ are derived from those given for geodesics by Vincenty (1975), for the simplified case of the meridian as a geodesic. Following his notation, the simplification can be made assuming a geodesic going from point ${P}_{1}$ on the equator to point ${P}_{2}$ at the station. The first step is to compute the following constants:

29 $$A=1+\frac{{e}^{{\prime}2}}{16384}\left\{4096+{e}^{{\prime}2}\left[-768+{e}^{{\prime}2}\left(320-175{e}^{{\prime}2}\right)\right]\right\}$$

30 $$B=\frac{{e}^{{\prime}2}}{1024}\left\{256+{e}^{{\prime}2}\left[-128+{e}^{{\prime}2}\left(74-47{e}^{{\prime}2}\right)\right]\right\}$$

Then compute the angular distance ${P}_{1}{P}_{2}$ on the sphere, which in this special case is simply the reduced latitude of the station:

31 $$U={\text{arctan}}\left[\left(1-f\right){\text{tan}}\,\phi \right]$$

Now compute the following angular correction:

32 $$\begin{aligned}\Delta U&=B\,{\text{sin}}\,U\,{\text{cos}}\,U\,\bigg\{1+\frac{1}{4}B\bigg[\left(-1+2{{\text{cos}}}^{2}\,U\right)\\ &\quad -\frac{1}{6}B\left(-3+4{{\text{sin}}}^{2}\,U\right)\left(-3+4{{\text{cos}}}^{2}\,U\right)\bigg]\bigg\}\end{aligned}$$

Finally, compute the geodesic distance $s$, which in this case is the northing:

33 $$N\left(\phi \right)=s=a\left(1-f\right)A\left(U-\Delta U\right)$$

To verify the computation, here are a couple of precise values for meridian arc length:

34 $$ \begin{gathered} N\left( {\pi /2} \right) = 10 001 965.72923\;{\text{m}} \hfill \\ N\left( {\pi /4} \right) = 4 984 944.37786\;{\text{m}} \hfill \\ \end{gathered} $$

### Numerical integration to test computation of northing $N(\phi )$

Whereas easting computation is an exact closed-form solution, the northing is fundamentally an ellipsoidal integral with no exact solution. That is, the above algorithm is an approximation. To test how good the approximation is, the method was compared to numerical integration, for which the step size can be adjusted until sufficient convergence is achieved. An appropriate method with flexible number of nodes is the alternative extended Simpson’s rule (Press et al. 1986) applied to the integral of the meridian radius of curvature ${R}_{M}\left(\phi \right)$:

35 $$\int\limits_{0}^{\phi }{R}_{M}\left(\phi ^{\prime}\right)d\phi ^{\prime}\approx \frac{\phi }{48m}\left[17{R}_{M}\left({\phi }_{0}\right)+59{R}_{M}\left({\phi }_{1}\right)+43{R}_{M}\left({\phi }_{2}\right)+49{R}_{M}\left({\phi }_{3}\right)+48\sum_{k=4}^{n-4}{R}_{M}\left({\phi }_{k}\right)+49{R}_{M}\left({\phi }_{m-3}\right)+43{R}_{M}\left({\phi }_{m-2}\right)+59{R}_{M}\left({\phi }_{m-1}\right)+17{R}_{M}\left({\phi }_{m}\right)\right]$$

36 $$\text{where }{R}_{M}\left({\phi }_{k}\right)= \frac{a(1-{e}^{2})}{{\left(1-{e}^{2}{{\text{sin}}}^{2}{\phi }_{k}\right)}^{3/2}}$$

The meridian from the equator to station latitude $\phi$ is divided into $m$ equal subintervals of length $\Delta \phi =\phi /m$ with nodes ${\phi }_{k}=k\Delta \phi$, $k=\left(\mathrm{0,1},\dots , m\right)$. For testing purposes, we start by specifying an approximate subinterval length $\widetilde{\Delta \phi }$, from which the number of subintervals is computed by rounding $m={\text{int}}(\phi /\widetilde{\Delta \phi })$. Then it is required that the minimum value of $m$ is 8. Since the numerator of ${R}_{M}\left({\phi }_{k}\right)$ is constant, it is taken outside of the integral and multiplied at the end.

An appropriate subinterval length is one for which the result does not change significantly when decreasing the length. Numerical experimentation shows that setting $\widetilde{\Delta \phi }=0.0005$ radians (with up to $m=3141$), gives no change to within 10 nm. It will be assumed in Sect. 3.4 that this gives us a ground truth for comparison of northing.

### Inverse transformation from GD $\left(E, N, h\right)$ to CG $(\phi , \lambda , h)$

For completeness, the inverse transformation is now presented, using GD $\left(E, N, h\right)$ coordinates to reconstruct CG $(\phi , \lambda , h)$ coordinates. As such an inversion would generally be rarely needed (except for testing), it is not compelling to seek an efficient non-iterative solution. Inversion from northing $N$ to latitude $\phi$ begins by iterating the following two formulas until there is negligible change in reduced latitude $U$, that is $\left|\delta U\right|<{10}^{-15}$ rad, starting with $\Delta U=0$:

37 $$U=\frac{N}{a\left(1-f\right)A}+\Delta U$$

38 $$\begin{aligned}\Delta U&=B\;{\text{sin}}\;U\;{\text{cos}}\;U\bigg\{1+\frac{1}{4}B\bigg[\left(-1+2{{\text{cos}}}^{2}U\right)\\ &\quad -\frac{1}{6}B\left(-3+4{{\text{sin}}}^{2}U\right)\left(-3+4{{\text{cos}}}^{2}U\right)\bigg]\bigg\} \end{aligned}$$

Typically, there are 6 iterations. Then geodetic latitude is given by:

39 $$\phi ={\text{arctan}}\left[\frac{1}{\left(1-f\right)}{\text{tan}}\;U\right]$$

Now, knowing the latitude, we can invert easting $E$ for longitude $\lambda$ in degrees:

40 $$\lambda ={\lambda }_{0}+\frac{180}{\pi {R}_{N}\left(\phi \right){\text{cos}}\;\phi }E$$

where radius of curvature of the prime vertical ${R}_{N}\left(\phi \right)$ was given previously. Note that the inversion requires reference longitude ${\lambda }_{0}(n)$ or equivalently, zone number $n$. Therefore, reference longitude (or zone number) should always be listed along with easting at every epoch in any specified file format. Inversion also requires that both easting and northing be provided together. Height is independent of northing and easting because they are defined on the ellipsoid at zero height, though it is convenient to provide height to complete the triplet of coordinates.

For completeness, a file format should specify station name, epoch time, reference longitude, easting, northing, and height, formal errors for easting, northing, height, and three correlation coefficients (given in Sect. 2.8). It is also convenient to include the assumed antenna height.

### Computation of formal errors and correlation coefficients for GD $\left(E, N, h\right)$

The GD formal covariance matrix is computed in the same manner as for TC coordinates, except it is computed at every epoch with a GC solution. The GC covariance matrix is rotated using latitude $\phi$ and longitude $\lambda$ also computed at every epoch:

41 $$\left(\begin{array}{ccc}{\sigma }_{E}^{2}& {\sigma }_{EN}& {\sigma }_{EH}\\ {\sigma }_{EN}& {\sigma }_{N}^{2}& {\sigma }_{NH}\\ {\sigma }_{EH}& {\sigma }_{NH}& {\sigma }_{H}^{2}\end{array}\right)=\mathbf{R}\left(\begin{array}{ccc}{\sigma }_{x}^{2}& {\sigma }_{xy}& {\sigma }_{xz}\\ {\sigma }_{xy}& {\sigma }_{y}^{2}& {\sigma }_{yz}\\ {\sigma }_{xz}& {\sigma }_{yz}& {\sigma }_{z}^{2}\end{array}\right){\mathbf{R}}^{{\text{T}}}$$

The formal errors are ${\sigma }_{E},{\sigma }_{N},{{\text{and}} \, \sigma }_{H}$. The correlation coefficients are:

42 $$ \begin{gathered} \rho_{EN} = \sigma_{EN} /\sigma_{E} \sigma_{N} \hfill \\ \rho_{EH} = \sigma_{EH} /\sigma_{E} \sigma_{H} \hfill \\ \rho_{NH} = \sigma_{NH} /\sigma_{N} \sigma_{H} \hfill \\ \end{gathered} $$

## Results

### Accuracy of GC $(x,y,z)$ to CG $\left(\phi , \lambda , h\right)$ by closure tests

First, we test the accuracy of computation of CG $\left(\phi , \lambda , h\right)$ from GC $\left(x,y,z\right)$ presented in Sect. 2.1. Accuracy is possible to test absolutely, because the inverse transformation from GC $(x,y,z)$ from CG $\left(\phi , \lambda , h\right)$ is exact. Since longitude is exact, it was set to zero for purposes of testing. The test proceeds as follows:

1. Specify CG $\left(\phi , \lambda , h\right)$ with known values for a variety of latitudes and heights.
2. Compute GC $(x,y,z)$ from CG $\left(\phi , \lambda , h\right)$ using Eq. (4).
3. Compute CG $\left(\phi ^{\prime}, \lambda ^{\prime}, h^{\prime}\right)$ from GC $(x,y,z)$ using the method of Sect. 2.1.
4. Latitude accuracy is defined as the maximum value of $\left|{\phi }^{\prime}-\phi \right|$.
5. Compute GC $\left({x}^{\prime},{y}^{\prime},{z}^{\prime}\right)$ from CG $\left(\phi^{\prime}, \lambda^{\prime}, h^{\prime}\right)$ using Eq. (4).
6. 3-D accuracy is defined as the maximum value of $\sqrt{{\left({x}^{\prime}-x\right)}^{2}+{\left({y}^{\prime}-y\right)}^{2}+{\left({z}^{\prime}-z\right)}^{2}}$.

There was no detectable latitude error. Maximum latitude errors are ${1\times 10}^{-15}$ rad for any point on Earth or in outer space, which is an upper limit, given that the results are at the quantization limit of double-precision computations. For heights of up to 500 km, the maximum 3-D error is 1 nm. At low-Earth orbiter heights around 1000 km, the maximum 3-D error is 2 nm. At GPS orbit heights around 20,000 km, the maximum 3-D error is 5 nm.

### Comparison of GC $(x,y,z)$ to CG $\left(\phi , \lambda , h\right)$ errors with Bowring

We compare 3-D errors of our proposed method of Sect. 2.1 with Bowring (1976) and Bowring (1985). In all methods including ours, Eq. (10) for height originally proposed by Bowring (1985) is used. 3-D errors are computed using the method of Sect. 3.1. Table 1 presents the comparison at various latitudes and heights. Approaching the equator and the poles, all methods give errors that tend toward zero in those limits, so these are not shown.

**Table 1:** Maximum 3-D Error for Methods of Bowring and of this paper

CG coordinates		3-D error (nm)		
Latitude	Height (m)	Bowring (1976)	Bowring (1985)	Blewitt
1$5^\circ$	1000	2	440	0
1$5^\circ$	4000	18	440	0
1$5^\circ$	10000	110	430	0
1$5^\circ$	40000	1800	420	0
30^∘^	1000	6	1500	1
30^∘^	4000	93	1500	1
30^∘^	10000	580	1500	0
30^∘^	40000	9100	1400	0
$45^\circ$	1000	9	1000	0
$45^\circ$	4000	140	1000	1
$45^\circ$	10000	890	1000	0
$45^\circ$	40000	14000	980	1
60^∘^	1000	6	170	0
60^∘^	4000	93	170	0
60^∘^	10000	580	170	0
60^∘^	40000	9200	160	0
75^∘^	1000	1	3	1
75^∘^	4000	18	3	1
75^∘^	10000	110	3	1
75^∘^	40000	1800	3	0

For Earth-bound heights, the method presented here can be considered practically exact (1 nm) and up to 3 orders of magnitude more accurate than the popular methods of Bowring (1976) and Bowring (1985). Not surprisingly, the same level of improvement is observed for latitude.

### Test of inversion: GD $\left(E, N, h\right)$ to GC $(x,y,z)$

Knowing that Earth-bound CG $\left(\phi , \lambda , h\right)$ are accurate to 1 nm for terrestrial positions, we can now test the precision of GD $\left(E, N, h\right)$ computations. Applying a similar method to Sect. 3.1, a variety of latitudes were selected at 10,000 km height and at zero longitude, and GC $(x,y,z)$ coordinates were computed exactly. GD $\left(E, N, h\right)$ coordinates were then computed and then inverted back to GC $(x^{\prime},y^{\prime},z^{\prime})$ coordinates. The 3-D error was then computed the GC coordinate differences.

Our results show that the magnitudes of differences were all < 2 nm, at the limits of double-precision computation. Note that this tests computational precision, and the ability to reconstruct the original GC $(x,y,z)$ time series. In contrast, testing the accuracy requires comparison with a known standard, for which we will use numerical integration in Sect. 3.4.

### Comparison of GD northing $N$ with numerical integration

The numerical accuracy of the GD northing $N$ computation of Sect. 2.5 is tested by comparison with the numerical integration method presented in Sect. 2.6. There it was verified that numerical integration converges to within 10 nm when decreasing the subinterval length to $\widetilde{\Delta \phi }=0.0005$ radians (up to 3141 subintervals). After testing many latitudes, the magnitude of the difference between computed GD northing coordinates and numerical integration results never exceeds 0.002 mm. This is consistent with (and smaller than) the level of accuracy claimed by Vincenty (1975), who noted that of all geodesic distances, north–south lines have the largest error. For all practical purposes, this level of error is insignificant, keeping in mind that this is the arc distance all the way to the equator (as much as 10 000 000 m).

### Comparison of GD $\left(E,N,h\right)$ and TC $\left(e,n,u\right)$ for polar time series

We now test whether our easting coordinate system shows systematic effects in practice for stations in arctic regions, owing to the coordinate singularity at the poles. For this, we choose the closest station to a pole that sits on bedrock (that is, station motion is representative of the solid Earth surface). That station is called HOWE in Antarctica, which is at $=-87.4^\circ, \;\lambda =-149.4^\circ ,\; \mathrm{and\; height }\;2582\;\mathrm{ m}$, with a span of 10.1 years of data, and 3471 daily epoch solutions in the time series. Whereas longitudinal zones are 11 km wide at the equator, at station HOWE they are only 0.5 km wide. Note that both GD and TC suffer from the polar singularity and TC cannot be considered a ground truth. The idea is that this test can tell us something about the magnitude of systematic effects.

The GD $\left(E,N,h\right)$ and TC $\left(e,n,u\right)$ time series were differenced; then, the average difference was subtracted from each epoch. Table 2 shows the statistics of these differences in millimeters.

**Table 2:** Differences between time series in GD and TC for station HOWE

	Easting (mm)	Northing (mm)	Height (mm)
RMS difference	0.007	0.007	0.000
Minimum difference	− 0.011	− 0.015	− 0.001
Maximum difference	0.018	0.013	0.000

The results show the root-mean-square (RMS) coordinate difference is less than 0.01 mm, and the worst-case differences have magnitudes below 0.02 mm. As an additional test, station velocity for each coordinate type was estimated by the robust algorithm MIDAS (Blewitt et al. 2016). The differences in estimated velocity were 0.002 mm/yr for easting, 0.002 mm/yr for northing, and 0.001 mm/yr for height.

### Comparison of GD $\left(E,N,h\right)$ and TC $\left(e,n,u\right)$ for large northward displacements

Now we test for predicted systematic differences between GD easting and TC east $\left(E-e\right)$ time series that arises from northward displacement. For this test, we go to Australia, which has some of the largest tectonic velocities in the northward direction. Stations with the longest history test the largest total northward displacements. Station TIDB, which is at $=-35.4^\circ,\; \lambda =-211.0^\circ ,\; \mathrm{and \;height }\;665\;\mathrm{ m}$, has a span of 28.1 years of data, with 9197 daily epoch solutions in the time series. Total northward displacement is 1.57 m. To investigate how such large northward tectonic displacement affects easting, GD $\left(E,N,h\right)$ and TC $\left(e,n,u\right)$ time series were differenced and statistics are shown in Table 3.

**Table 3:** Statistics of differences between GD and TC time series for station TIDB

	Easting (mm)	Northing (mm)	Height (mm)
RMS difference	0.099	0.000	0.044
Minimum difference	− 0.169	− 0.001	− 0.075
Maximum difference	0.319	0.001	0.090

Differences in northing are completely negligible. Differences in height are < 0.1 mm over 3 decades. As predicted, differences in easting are largest, with RMS difference of 0.1 mm, and maximum difference of 0.3 mm (again, over 3 decades). As might be expected, the differences in the easting have an obvious systematic trend, given that the station is steadily moving with a northward velocity.

The MIDAS algorithm (Blewitt et al. 2016) was applied to both types of time series, in order to quantify the trend difference in easting. The difference is 0.013 mm/yr, which can be considered negligible when compared to other sources of error.

## Discussion

### Significance of results

Section 2.1 presents an improved method of computing CG $\left(\phi , \lambda , h\right)$ from GC $(x,y,z)$ based on a proposed improvement to the equation of reduced latitude for subsequent input into the equation of latitude of Bowring (1976). Test results show that the improved method is practically exact, with maximum errors of 1 nm for Earth-bound positions, and errors in latitude no greater than ${10}^{-15}$ rad for all points on the Earth and in outer space. These numbers reflect accuracy, since the forward transformation is exact and so the inverse provides an absolute test.

The test of GD $\left(E,N,h\right)$ inversion back to GC $(x,y,z)$ shows that the forward and inverse computations of coordinates are self-consistent to within 2 nm. Our test of the northing computation shows it is consistent with numerical integration to within $2\; \mu {m}$, which therefore is our upper-limit estimate of the accuracy of GD computation.

All the test results show that differences (after subtraction of the mean) between time series of GD $\left(E,N,h\right)$ and TC $\left(e,n,u\right)$ are insignificant, with trends less than 0.02 mm/yr. For the longest running time series spanning 3 decades with maximum northward tectonic displacement, RMS epoch differences are less than 0.1 mm. Differences between GD and TC can be expected because GD incorporates Earth curvature. Interpretation of coordinates starts to breakdown for displacements exceeding 30 m, which almost never falls within the limits of maximum displacement for contemporary measurement of solid Earth deformation. We can therefore treat relative coordinate time series from GD and TC systems as equivalent for purpose of scientific interpretation of station displacement for most types of geodetic investigations of geophysical displacement.

### Examples using the global information content in GD $\left(E,N,h\right)$

GD $\left(E,N,h\right)$ inherently retain information on the global geodetic location of the station with respect to the ellipsoid. This can be used to advantage in two important situations.

The first tangible benefit was realized by Argus et al. (2011). In that publication, station time series from historical stations were suspected to be on or near the same reference point as modern station benchmarks. The GD $\left(E,N,h\right)$ made it possible to directly compare different station time series without special analysis, as it became obvious whether both time series could be tied into one, thus effectively extending time series for site velocity estimation. GD $\left(E,N,h\right)$ made it very simple to tie time series together. In the case of TC $\left(e,n,u\right),$ such functionality is not possible with separate reference points for each station, and ad hoc TC time series generation is required with single reference points in a cluster of stations.

This benefit could also be applied when intentionally decommissioning a station and running a replacement station side-by-side for a period that allows for the two series to be merged. Coordinates from GD $\left(E,N,h\right)$ make this extremely easy, in contrast to TC $\left(e,n,u\right)$, which would require a special analysis to determine both station time series using an identical reference Cartesian coordinate.

Another tangible benefit came to light over the last decade with the ever-expanding GPS network, which now approaches 20,000 stations (Blewitt et al. 2018). It turns out to be a frequent problem that two different stations can have the same name. This can lead to accidental merging of different time series, often with stations on different continents. In this case the TC $\left(e,n,u\right)$ system saturates, as the reference point on a different continent is too far away for the relative coordinate to be interpreted (or worse, the format may be numerically saturated). With GD $\left(E,N,h\right)$ system that never happens, because curvature of the Earth is accounted for, and coordinates directly correspond to latitude and longitude. GD $\left(E,N,h\right)$ coordinates are useful for debugging when different time series are accidentally merged.

## Conclusions

An improved non-iterative computation of latitude has been presented based on Bowring (1976) but with a different equation for reduced latitude. The method proves to be practically exact for geodetic applications for any set of GD coordinates, terrestrial or outer space. It is noted that double-precision computations limit the testing of the true accuracy, so ${10}^{-15}$ rad should be considered an *upper* limit on latitude accuracy. Without iteration, the method improves the commonly used methods of Bowring (1976) and Bowring (1985) by typically 3 orders of magnitude.

A graticule distance (GD) coordinate $\left(E,N,h\right)$ system has been presented with sufficient detail for users to write software to compute the coordinates, given geocentric Cartesian (GC) coordinates $(x,y,z)$, and to reproduce the results shown here. Demonstrated here is that GD $\left(E,N,h\right)$ precisely reproduce time series of relative coordinates $\left(e,n,u\right)$ from the traditional topocentric Cartesian (TC) system for the case of stations that move with tectonic motions over decadal time scales. The differences between GD and TC time series are negligible, with maximum drifts of 0.01 mm/yr.

Unlike TC coordinates $\left(e,n,u\right)$ which are inherently local and relative, GD coordinates $\left(E,N,h\right)$ retain all global information content, allowing for capabilities that do not exist for TC. Capabilities include (1) the ability to relate position of stations closer than a few km with GD coordinates, with benefits such as merging of time series for geophysical interpretation, or identifying which benchmark was used for a historical station name, and (2) the ability to identify and debug blunders when two time series of the same station name are accidentally merged, when they refer to physically different benchmarks.

The disadvantage of the traditional TC $\left(e,n,u\right)$ system is that, operationally, each GNSS station has a different reference point for TC coordinates, hindering the ability to directly compare time series from different stations without applying ad hoc methods. Given that there is no significant extra cost to implement a GD $\left(E,N,h\right)$ system, transition from TC to GD should be seamless for most geodetic applications.

It is noted that since 2011, Nevada Geodetic Laboratory has publicly produced time series for more than 20,000 GPS stations in GD $\left(E,N,h\right)$ form that have been cited by hundreds of studies (Blewitt et al. 2018). That testifies to widespread adoption of the GD system, which is for the first time described in sufficient detail here to enable reproduction of the results presented.

## Data Availability

GPS time series data used by this study for testing are available from the Nevada Geodetic Laboratory repository, http://geodesy.unr.edu/. All input parameter values used for testing are given in the article along with the results.
